# Neuronal activity underlying vocal production in bats

**DOI:** 10.1111/nyas.15410

**Published:** 2025-07-21

**Authors:** Susanne S. Babl, Ava Kiai, Francisco García‐Rosales, Julio C. Hechavarría

**Affiliations:** ^1^ Brain and Behavior Group Ernst Strüngmann Institute for Neuroscience in Cooperation with the Max Planck Society Frankfurt Germany; ^2^ Institute of Cell Biology and Neuroscience Goethe University Frankfurt Frankfurt Germany

**Keywords:** bats, echolocation, neural circuits, social communication, vocal production

## Abstract

Bats exhibit a unique repertoire of vocal behaviors, with many species employing echolocation to actively sense their environment while using communication calls for social interactions. This review explores the neural circuits underlying these behaviors, from brainstem and midbrain networks that generate and control vocalizations to higher order brain regions, such as the frontal cortex, which may contribute to the modulation of vocal behaviors. Although much is known about brainstem mechanisms for echolocation, less is understood about the neural control of communication calls and the integration of these systems. Recent findings highlight the interplay between auditory, motor, and spatial processing networks in shaping bat vocalizations, with evidence for both shared and distinct neural pathways for echolocation and communication. We propose a framework for vocal production circuits in bats, synthesize findings from diverse species and experimental techniques, and identify key questions to guide future research. This review underscores the importance of bats as models for studying vocal production and how they can provide insights into the evolution and adaptation of neural mechanisms across species.

## Graphical abstract

Bats are known for their highly evolved echolocation and social vocalizations. They have been well studied in terms of auditory processing, but their neural circuits for vocal production are far less understood. This review highlights recent progress in mapping neural activity linked to vocalization in bats. We identify key neural hubs for generating and controlling vocalizations and outline open questions to guide future research on vocalization circuits in bats and other mammals.

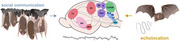

## INTRODUCTION

**FIGURE 1:**
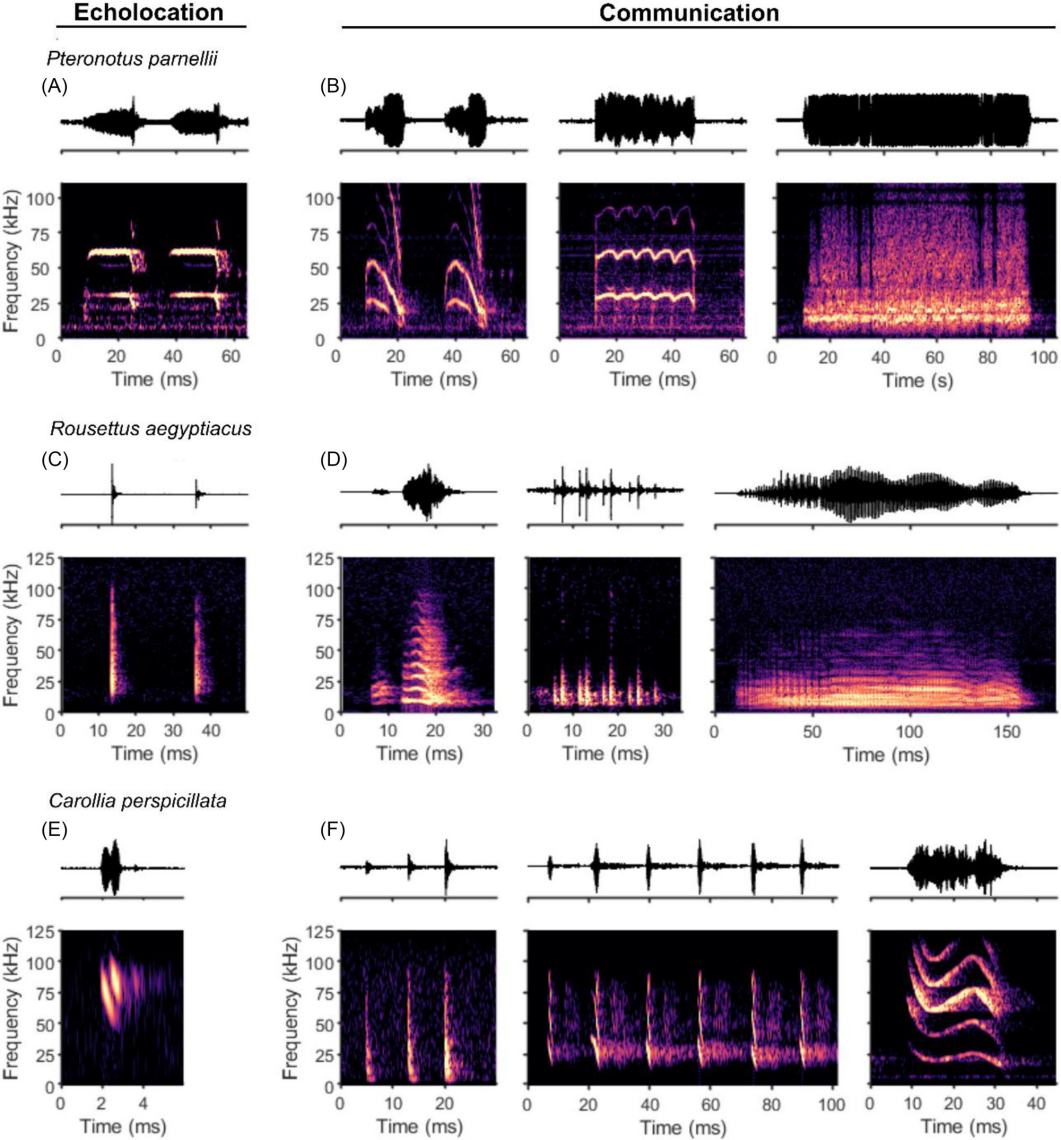
Examples of echolocation and communication calls of three bat species from different families. (A–B) *Pteronotus parnellii*. (A) Oscillogram (top) and spectrogram (bottom) of an echolocation call with constant frequency (CF) and frequency‐modulated (FM) components and (B) a selection of communication calls, namely, sHFM (left), dRFM (middle), and rBNB (right). (C–D) *Rousettus aegyptiacus*. (C) Oscillogram (top) and spectrogram (bottom) of echolocation pulse produced with tongue click and (D) a selection of communication calls. (E–F) *Carollia perspicillata*. (E) Oscillogram (top) and spectrogram (bottom) of echolocation call with FM component and (F) a selection of communication calls recorded from the laboratory‐housed colony. *Source*: (A and B) Examples reproduced with permission from Jagmeet Kanwal, Georgetown University; (C and D) examples reproduced from the public dataset available at Figshare (https://doi.org/10.6084/m9.figshare.c.3666502.v2) by Ref. 166.

Bats are highly vocal animals, and the majority of species in this diverse order (comprising over 1400 species) use their vocalizations for two main and quite distinct purposes. First, many bats produce echolocation calls to actively sense their environment by processing the returning echoes from their surroundings. Second, bats also emit diverse social communication calls (Figure 1), with some species producing complex songs^1^, ^2^ and showing evidence for vocal learning.^3^, ^4^

In general, bats live in an astounding variety of ecological niches and have come to exploit nearly all possible food sources in nature (such as insects, pollen, fruit, nectar, and blood^5^). The design of ultrasonic echolocation pulses has likewise come to vary widely between bat species, reflecting the fine‐tuning of each species’ sensory and physical traits to its specific environment. The frequency composition, length, timing, and structure of calls may all differ across each species’ orientation signals. Although some bat species from the same family may exhibit remarkably different vocal adaptations in echolocation, such as members of the Vespertilionidae, other distantly related species have evolved strikingly similar echolocation calls.

Most bats emit short (<1–3 ms), downward frequency‐modulated (FM) sweeps,^6^ such as those in the genera *Myotis* or *Carollia* (Figure 1E). The high frequencies present in these calls make them especially suited for the detection of small objects, whereas their multi‐harmonic sweep structure enables bats to localize target position with great accuracy, even in cluttered environments.^7^

In addition, their short duration means that outgoing pulses and returning echoes are unlikely to overlap in time. However, overlaps may occur at short distances (<1 m) to objects and may interfere with the animal's ability to interpret information from the echo. To combat this, bats using FM echolocation will tend to adapt the pulse emission rate (as exemplified in the terminal buzz of the big brown bat, *Eptesicus fuscus* ^8^) and shorten pulse duration as they approach a target or when flying in cluttered environments.^6^, ^9^

In contrast, Old World horseshoe (*Rhinolophus* spp.) and Old World leaf‐nosed bats (*Hipposideros* spp.), as well as the New World mustached bat (*Pteronotus parnellii*), emit longer orientation signals that feature a prominent constant frequency (CF) component, in some cases flanked on one or both sides by a short FM component (CF‐FM echolocation, Figure 1A).^10^ As CF‐FM echolocation is less common than FM echolocation, and the species that employ it are split between the two bat suborders, this echolocation strategy may have evolved independently in each suborder—a possible example of convergent evolution.^6^, ^10^

By concentrating energy in a narrow frequency band, CF‐FM echolocation pulses may provide a reliable signal over longer distances and focus on information about target motion.^10^ The fluttering movement of winged insects results in patterns of amplitude and frequency fluctuations in the CF component of returning echoes, aiding in predation and potentially allowing the bat to distinguish among different types of insects.^11^ While FM echolocators avoid interference between pulses and echoes by ensuring they do not overlap in time, CF‐FM echolocators maintain this distinction in the frequency domain.^10^ The Doppler shift phenomenon results in the CF component returning to the bat at a slightly higher frequency than the outgoing pulse. In addition, at least some CF‐FM echolocators are also known to actively adjust vocalizations to account for the Doppler shift (this strategy will be discussed in detail below^12^).

Importantly, these different echolocation design strategies each demand specialized neuroanatomical circuits to produce them and evaluate the relevant elements in the resulting echoes. These circuits will be discussed below to the extent that they are involved in signal production.

While bats are famous for their echolocation behavior, they also communicate using a large repertoire of vocalizations which are each deployed in specific social contexts (such as distress or during territorial combat,^13^ with some species showing highly specialized behaviors such as maternal use of infant‐directed vocalizations^14^). They are one of only few mammals that produce complex songs,^1^ such as the greater sac‐winged bat (*Saccopteryx bilineata*)^15^ and the Mexican free‐tailed bat (*Tadarida brasiliensis*), whose songs are composed of syllables and phrases, which are organized by recognizable syntactic rules.^16^ Bats are also one of only three known mammalian orders in which there is evidence of vocal learning.^17^, ^18^, ^19^, ^20^ Juvenile *S. bilineata* bats exhibit remarkably complex vocal development, which includes going through a *babbling* phase in which they attempt and gradually learn to produce adult‐like syllables.^21^

In other species, adults may emit social contact calls,^22^ and signal group membership through a vocal signature, analogous to human dialects.^3^, ^23^

The brain circuitry and neuronal activity underlying vocal behavior have been the subject of many studies over the past years. Although a large body of literature has focused on the role of brainstem networks in echolocation calls, much less is known about the neural control of communication calls, and in what way these brain circuits overlap or diverge. Furthermore, while recent studies have explored the role of higher order brain regions, such as the frontal cortex, in vocal production, our understanding of their specific contributions remains limited.

**FIGURE 2:**
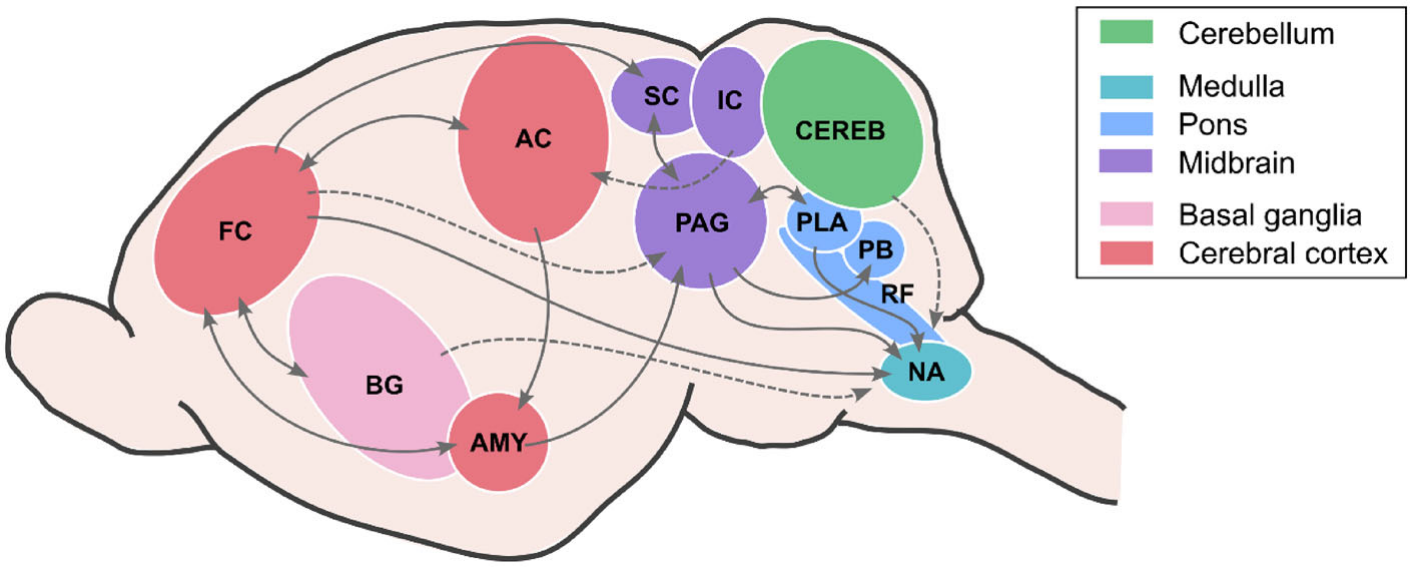
Schematic illustration of important brain structures in vocal production and their anatomical connections. Solid lines indicate direct projections, and dashed lines indicate indirect connections via further brain regions. AC, auditory cortex; AMY, amygdala; BG, basal ganglia; CEREB, cerebellum; FC, frontal cortex; IC, inferior colliculus; NA, nucleus ambiguus; PAG, periaqueductal gray; PB, parabrachial complex; PLA, paralemniscal area; RF, reticular formation; SC, superior colliculus.

In this review, we aim to bring together diverse findings from in vivo electrophysiological experiments, as well as pharmacological and electrical manipulations, which together seek to describe, perturb, and identify the neural underpinnings of executive motor control of vocal production. Taking a bottom‐up approach, we will discuss investigations of brain regions from brainstem circuits to the frontal cortex, highlight functional interactions with auditory and spatial processing, and propose a tentative pathway for vocal production in bats. The brain areas implicated in vocal production and control in bats are summarized in Figure 2, and they will be discussed in detail throughout this review. We emphasize that our current knowledge of bat vocalization is derived from diverse species models, techniques, and occasionally conflicting empirical findings, making it challenging to create a unified overview of the field. Our aim is to chart progress to date and highlight unresolved questions that can guide future studies of vocal production behavior in bats and other animal models.

## VOCAL CONTROL IN BRAINSTEM AND MIDBRAIN CIRCUITS

### Laryngeal output and its control in the medulla

Nearly all echolocating bats use the larynx to produce echolocation calls. The primary exceptions are bats within the genus *Rousettus*, which perform tongue clicks to emit broad‐spectrum sonar pulses and use the larynx only for communication calls (Figure 1C).

Sounds used for the detection and localization of objects must have certain properties: They must have relatively short wavelengths (i.e., have a high frequency), in order for the sound wave to reflect off the surface of small objects;^24^ and be short in duration to permit the computation of object location in space, particularly for proximate objects.

The bat larynx exhibits several evolutionary adaptations to meet the demands of echolocation, such as ossified cricoid and thyroid cartilages and exceptionally fast laryngeal muscles.^25^, ^26^ These specializations make it possible to produce high‐frequency echolocation pulses with call durations and inter‐pulse intervals in the millisecond or even sub‐millisecond range, while still allowing for the production of communication calls in lower frequencies and on longer time scales. Some bat species, such as Daubenton's bats (*Myotis daubentonii*) of the Vespertilionid family, can expand their frequency range to up to seven octaves, producing the lowest frequency portion by recruiting ventricular folds and achieving exceptionally high frequencies through the use of specifically adapted vocal membranes.^27^

In the mammalian vocal system, laryngeal muscles are innervated by motor neurons that are in turn controlled by structures within the brainstem. The nucleus ambiguus (NA, see list of abbreviations in Table 1) within the medulla has attracted special attention in the search for the neural basis of vocal production as it presents the architecture required for vocal motor control. It projects to motor neurons that, in addition to the laryngeal muscles, also control supralaryngeal articulator muscles and expiration^28^, ^29^ and is innervated by the periaqueductal gray (PAG, see below). In at least one bat species, a direct projection from the motor cortex to the NA has been identified, namely, in the Egyptian fruit bat (*Rousettus aegyptiacus*),^30^ which uses its larynx for social communication, though not for echolocation calls.

**TABLE 1:** List of abbreviations

Abbreviations	
AC	auditory cortex
ACC	anterior cingulate cortex
BG	basal ganglia
CF	constant frequency
CF‐FM	constant frequency – frequency‐modulated
CN	caudate nucleus
FAF	frontal auditory field
FM	frequency‐modulated
IC	inferior colliculus
LFP	local field potential
NA	nucleus ambiguus
PAG	periaqueductal gray
PLA	paralemniscal area
SC	superior colliculus

Electrophysiological recordings of the NA of the rufous horseshoe bat (*Rhinolophus rouxii*) revealed neuronal spike patterns time‐locked to onsets or offsets of their CF‐FM echolocation calls. NA neuron firing started tens of milliseconds before vocalization onset and abruptly stopped a few milliseconds before the end of the calls, and firing rates were positively correlated with the emitted call frequency.^31^ It is an open question whether NA neurons track call durations or other properties of echolocation calls (such as frequency) in bat species with shorter FM echolocation signal designs as well as in CF‐FM echolocators.

### The PAG and pontine nuclei

**FIGURE 3:**
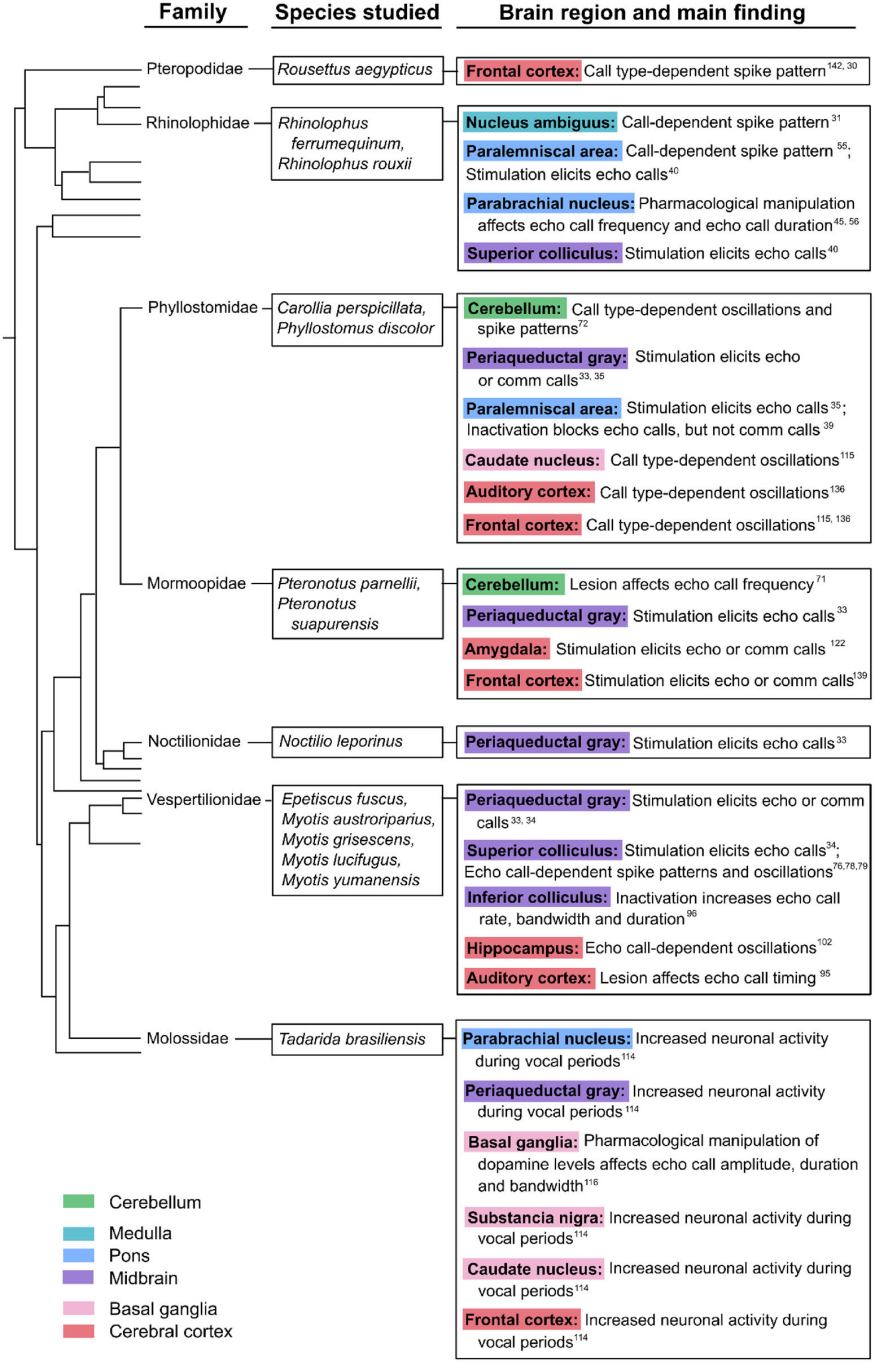
Research on the neuronal network of vocal production mapped to the bat cladogram showing the investigated species, the studied brain region, and a summary of the main findings for each family. Superscript numbers indicate corresponding references in the reference list. comm, communication calls; echo, echolocation calls.

Upstream of the NA (Figure 2), neurons in the bat PAG play a key role in the control and gating of innate vocalizations, as has been widely demonstrated in rodents, cats, and primates.^28^, ^29^, ^32^ Several studies investigating different bat species have demonstrated that stimulation in the PAG elicits vocalizations (see Figure 3 for an overview).

When Suga et al. electrically stimulated the lateral PAG in species of phyllostomids, noctilionids, and vespertilionids—among them *E. fuscus*—all bats uniquely produced echolocation calls that were, in all cases, similar to their species‐specific echolocation signal.^33^ In contrast, when Valentine et al. electrically stimulated the lateral PAG of *E. fuscus*, they only observed the production of communication calls.^34^ However, both echolocation and a suite of communication calls were observed following electrical stimulation to the lateral and ventro‐lateral areas of the caudal PAG in the pale spear‐nosed bat (*Phyllostomus discolor*).^35^

Taken together, these results may suggest that these two vocalization regimes in bats are controlled by distinct regions within the vocally selective portions of the PAG, as has been demonstrated in primates^36^ and cats.^37^ However, as it is difficult to compare the precise regions targeted across these studies and especially challenging to do so between species, it is also possible that the bat PAG features spatial overlap of call type–specific neural populations, as evidenced in mice,^32^ or even that there are subtle differences between bat species with regard to the functional design of the PAG.

The PAG communicates extensively with neighboring structures within the dorsal pontine tegmentum, such as the paralemniscal area (PLA).^38^ When neuronal activity in this brain region is blocked pharmacologically, stimulation in the PAG can no longer elicit echolocation calls, but PAG‐induced communication calls are still observed in *P. discolor*.^39^ By comparison, pharmacological or electrical stimulation of the PLA alone leads only to the production of echolocation calls, but never communication calls in rhinolophid and phyllostomid bats.^35^, ^40^

Adjacent to the PLA, the lateral lemniscal nuclei, embedded within axons projecting from the cochlear nucleus to the inferior colliculus (IC), may contribute to vocal control by distinguishing self‐generated from exogenous sounds early in the ascending auditory pathway. Recording in the nuclei of the lemniscus, Suga and Schlegel found that evoked responses to self‐generated vocalizations were attenuated as compared with those evoked from playback of those same vocalizations, a distinction not present at the level of the auditory nerve.^41^, ^42^ The inhibition of self‐generated sounds is critical in the case of the echolocating bat. Although other animals that communicate vocally must also deal with self‐stimulation, bats risk becoming desensitized to returning echoes if auditory‐responsive neurons are already engaged in responding to their own vocalizations.

Caudal to the PLA is the parabrachial complex, a group of nuclei mainly serving as a relay of cardiovascular, gastrointestinal, and respiratory information between the medulla and higher structures.^43^ As bats vocalize during the expiration phase of a breath,^44^ it follows that the vocal pathway is tightly linked with respiratory control.

When Smotherman et al. enhanced GABAergic (i.e., inhibitory; see Glossary in Table 2) synaptic activity in the parabrachial complex of the greater horseshoe bat (*Rhinolophus ferrumequinum*), they not only observed longer respirations but also prolonged echolocation call durations. In contrast, when GABAergic synaptic activity was blocked, the breaths became shorter, and the animals were no longer able to accommodate two echolocation calls in one expiration, a typical behavior in this animal.^45^

**TABLE 2:** Glossary

Glossary	
Coherence	A measure of synchrony that relies on the phase relationship and consistency of oscillations.
Dopaminergic neurons	Neurons releasing the neurotransmitter dopamine. In the central nervous system, they are mostly located in the substantia nigra and the ventral tegmental area and play a critical role in movement control, reward and motivation.
Echo‐delay tuning	The sensitivity exhibited by some neurons to specific temporal delays between the onset of an outgoing echolocation call and the incoming echo. Neurons that exhibit echo‐delay (or more simply, delay) tuning fire preferentially after the arrival of the echo at their preferred delay.
GABAergic neurons	Neurons which release gamma‐aminobutyric acid (GABA), the primary inhibitory neurotransmitter in the mammalian central nervous system. By inhibiting excitatory neurons in the brain, they can gate signal flow and contribute to rhythmic brain oscillations.
Glutamatergic neurons	Neurons which release glutamate, the primary excitatory neurotransmitter in the mature mammalian central nervous system.
Local field potential	A continuous electrical signal generated by extracellular potentials of populations of neurons and believed to represent the sum of synaptic input at the recording site. LFPs are typically decomposed into particular frequency bands, named roughly in the order in which rhythmic activity in these bands were first observed: delta (0.1‐4 Hz), theta (4‐8 Hz), alpha (8‐12 Hz), beta (12‐30 Hz) and gamma (30‐120 Hz).
Neurotransmitter agonist/antagonist	A substance which binds to a synaptic receptor and causes its activation, in the case of agonists – thus mimicking the behavior of endogenous neurotransmitters – or blocks its activation, in the case of antagonists.

Together with the above, these findings indicate that the midbrain and brainstem nuclei perform a suite of complex operations that range from the switching on and off of vocalization to fine‐tuned control of complex vocal behaviors that crucially depend on sensory feedback. PAG‐generated signals for echolocation depend on intact neuronal activity patterns in the PLA to achieve the corresponding motor action in the larynx, whereas PAG signals for communication calls may act through alternative pathways. Meanwhile, pontine structures medial to the lateral lemniscus are a site of convergence for multiple functions, serving the top‐down vocal‐motor output circuitry and bottom‐up auditory processing functions in parallel.

## THE AUDIOVOCAL INTERFACE: EXAMPLES FROM DOPPLER‐SHIFT COMPENSATING BATS

The proper control of vocalizations requires tight integration between sensory processing, premotor preparation, and motor execution circuits. When hearing is disrupted, as in deaf populations, it becomes difficult or impossible to control vocal frequency and loudness.^46^ A clear and elegant example illustrating the importance of auditory feedback and the role of the ascending auditory pathway in vocal production is the Doppler shift compensation behavior (for a review, see Ref. 12). In this section, we will describe findings from auditory brain regions as they pertain to this example of feedback‐dependent vocal control.

In 1968, Schnitzler discovered that *R. ferrumequinum* emit CF‐FM calls with a particular frequency while at rest (*resting frequency*) but will lower the frequency of the CF component during flight by up to 8 kHz^11^, ^47^ to compensate for flight‐induced as well as target movement‐induced Doppler shift of the returning echo.^48^ This compensation ensures that the echo returns to the bat at its *reference frequency*—the frequency at which the auditory system is most sensitive (see below^49^, ^50^). This adjustment of the echolocation frequency was named Doppler shift compensation and has been observed in several rhinolophid,^51^ hipposiderid,^52^ and mormoopid^53^ species with CF‐FM echolocation.

Appropriate adjustment of the CF call naturally requires auditory feedback that permits comparison between emitted pulses and received echoes. The PLA in Doppler‐shift compensating rhinolophid bats features different populations of vocally active neurons. Some neurons show suppression during vocalization, whereas other subpopulations of neurons ramp up activity in the pre‐vocal phase and/or maintain high firing rates for the duration of the vocalization. These vocally active neurons also exhibited echo‐delay tuning (see Glossary in Table 2), firing when an artificial echo was played at particular delays (5–18 ms) after the onset of the animal's own echolocation. Interestingly, this echo‐delay sensitivity was specific to trials in which the bat itself produced the echolocation call and not present when the bat simply heard an artificial echolocation call followed by an echo,^54^, ^55^ potentially allowing the bat to distinguish its own echo versus the echoes belonging to other bats. The vocal‐related inhibition seen here and the ability to distinguish self‐generated from heard echolocation sounds could be driven by inhibitory inputs from nuclei of the lateral lemniscus (discussed above).

In subsequent studies, systematic perturbation of the paralemniscus‐adjacent parabrachial nucleus using pharmacological agents targeting GABAergic and glutamatergic neuronal populations showed that local inhibitory activity was crucial both for maintaining the CF at the preferred frequency at rest and for performing the correct pitch compensation. When GABA antagonists or glutamate agonists were injected into the region, the resting frequency rose, and the bats could not lower the CF component in response to a playback that mimicked the Doppler shift induced by natural flight. Conversely, when GABA agonists or glutamate antagonists were applied, the resting frequency lowered, and the bats overcompensated for the Doppler shift, lowering CFs below levels observed in typical conditions.^56^

In these manipulations, CF‐FM echolocation calls were shifted in frequency as a whole. Only when the investigators injected the GABA antagonist into the principal sensory nucleus of the trigeminal region, ventral and caudal to the parabrachial nucleus, was the structure of the call itself perturbed: the spectral shape of the CF‐FM call was severely distorted, atypical amplitude modulations were introduced, and bats began producing calls in pairs of unequal durations.^56^

In Doppler‐shift compensating bats, the sensory system exhibits widespread adaptations to optimize the processing of CF‐FM signals. Virtually all stations along the auditory processing pathway exhibit exquisite sensitivity to the reference frequency (a reflection of an auditory fovea), originating in the cochlea and observable at the level of the basilar membrane,^57^, ^58^ the IC,^49^, ^59^, ^60^ and the auditory cortex (AC).^11^

In these bat species, the tonotopic plane of the primary AC is not uniformly responsive to sounds across all frequencies but features large expansions of the regions dedicated to the frequencies surrounding the reference frequency, even at exceptionally low sound levels.^11^

Overall, this widespread specialization of the neuroanatomy to produce and process signals in the foveal frequency range demonstrates how tightly integrated the neural machinery for the transmission and reception of specialized signals is. Meanwhile, the results from midbrain areas mentioned here illustrate that these subcortical areas perform specialized functions important for (1) computing necessary vocal adjustments following sensory input, (2) inhibiting population responses to self‐generated sounds while maintaining high sensitivity for returning echoes, and (3) maintaining the frequency and temporal structure of vocalizations even as they are being produced.

Of course, bats that do not exhibit Doppler‐shift compensation also possess remarkable vocal plasticity and can flexibly and rapidly adapt many aspects of their vocalizations,^61^ such as the frequency,^62^ timing,^63^ and amplitude—known as the Lombard effect.^64^, ^65^ Although previous reviews have sketched a putative circuit for feedback‐dependent vocal control for such behaviors,^66^ studies directly linking neural activity to vocal adaptations beyond Doppler‐shift compensation are still lacking.

## CEREBELLAR CONTRIBUTIONS TO VOCAL PRODUCTION

One of the best described functions of the cerebellum is its role in movement control and regulation, which it executes both through inputs to brainstem motor circuits and through projections to the motor cortex that run via the thalamus, as described in primates.^67^, ^68^ Its function in vocal control has rarely been investigated to date, but abnormal vocalizations after cerebellar lesions have been described in macaques (*Macaca fuscata*), and cerebellar disorders in humans can lead to deficits in speech production.^69^, ^70^

In bats, the involvement of the cerebellum in vocalizations was demonstrated many years ago by Horikawa and Suga through cerebellar lesions in *P. parnellii*, which resulted in an increased variation of the CF component in CF‐FM echolocation calls.^71^ A recent study from our research group on Seba's short‐tailed bats (*Carollia perspicillata*) revealed that cerebellar firing rates and local field potential (LFP) oscillation patterns differ depending on whether the bat produces an echolocation or a communication call^72^ (see Figure 1, e.g., calls in both bat species). These call type–dependent changes in activity patterns were already observed up to half a second before the onset of the vocalization, indicative of premotor activity.

## SPATIAL PROCESSING AND SOCIAL REPRESENTATIONS IN COLLICULAR CIRCUITS

### Echolocation fine‐tuning and read‐out in the superior colliculus

The mammalian superior colliculus (SC) has been implicated in spatial processing and orientation behaviors by integrating sensory information across different modalities, especially from the visual and acoustic domains.^73^, ^74^, ^75^ Neurons in the bat SC show clear delay tuning, thereby encoding target distance and creating an egocentric map of the environment.^75^, ^76^, ^77^ As the perception of spatial features is dependent upon echolocation call emissions, it seems that the bat SC has extended its function from controlling eye or head movements to the initiation and structuring of echolocation calls. When Valentine et al. electrically stimulated the SC in *E. fuscus*, they not only observed head and pinna movements but also could elicit stereotypical echolocation calls. These calls occurred after a significantly longer latency than vocalizations induced by stimulation of the PAG, indicative of a longer pathway to motor neurons.^34^

The production of echolocation calls through SC stimulation had already been shown in rhinolophid bats,^40^ where the authors found that the amplitude level or the duration of the elicited call increased with increasing stimulation current, a feature they did not observe when eliciting calls from any other brainstem area. Electrical stimulation with stronger current can both increase the firing rate in a given neural population or affect a larger volume of tissue thereby recruiting a larger population of neurons. It cannot be trivially disentangled whether it is specifically the neuronal firing rate, or the size of the active neural population (or a combination of both), that modulated echolocation call parameters. These findings do, however, point to an active role of the SC in shaping and adapting vocalizations depending on the current environmental conditions.

This function was further confirmed when neuronal activity was recorded in the SC of *E. fuscus* while the bat was tracking a moving insect.^78^, ^79^ Vespertilionid bats modify their FM call duration and interval depending on the distance to the target they pursue.^8^ When the target was further away, longer echolocation calls were produced, and neurons in the SC fired tens of milliseconds before the onset of the call. As the target approached, the calls and call intervals became shorter and neurons started spiking closer in time to the call onsets. Therefore, the spike latency of this premotor activity correlated with call duration and call interval.^78^, ^79^ The authors also observed pre‐vocal spike bursts (∼3 ms before onsets), whose latencies were independent of call duration or call interval.^78^ Thus, on the one hand, the SC prospectively encodes echolocation call parameters and a signal that could be sent downstream to control motor output. On the other hand, it signals the initiation of every echolocation call irrespective of its temporal characteristics, a potentially useful signal for priming the network for an incoming echo.

A follow‐up study in *E. fuscus* could show that delay tuning of SC neurons is also influenced by echolocation properties.^76^ While the bats were freely flying between obstacles and naturally increased their call rate as they approached certain objects, neurons in the SC shifted their preferred echo delay from longer to shorter time periods. These neurons also exhibited sharper tuning to these delays, thereby obtaining a higher spatial resolution at close range. Sharpening in delay tuning with increasing echolocation rate may, however, be independent of active vocal production as it was observed in the AC and IC to the playback of call–echo sequences versus single call–echo pairs.^80^, ^81^ High echolocation call rates may additionally be accompanied by an increase in the power of gamma oscillations [40–140 Hz] (see Glossary in Table 2) in the SC,^76^ a rhythmic activity pattern that is known to emerge when groups of neurons engage in attention tasks.^82^ This rhythmic activity pattern could also stem from sequences of evoked potentials triggered by echolocation calls produced at high rates.

There is also evidence for some functional mapping in the SC. Wohlgemuth et al. found that neurons in dorsal SC layers of *E. fuscus* are more active after an echolocation call (putatively processing sensory information), and neurons in ventral SC layers are primarily active shortly before call onset, suggesting premotor coding.^79^

Taken together, these findings implicate the midbrain SC as a powerful network where spatial information is not only analyzed, but where sampling of the environment may be optimized through dynamic adaptation of echolocation behavior—according to the animal's current position relative to targets and its need for finer spatial or temporal resolution.

### Contributions of the inferior colliculus

The IC, which is exposed on the dorsal brain surface in many bat species,^83^ is not usually considered a spatial processing site across mammals, but rather as a critical hub in the auditory system. Inputs from all auditory nuclei in the brainstem along the ascending auditory pathway converge in the IC. These inputs arrive both through ipsilateral and contralateral connections and therefore provide monaural and binaural information. IC output travels through the auditory thalamus to the AC, which in turn sends top‐down projections back to the IC.^84^ Neurons in the IC are tonotopically organized and often exhibit sharp frequency tuning curves.^85^, ^86^ In addition to frequency information, the mammalian IC also processes temporal properties of sound (e.g., via duration‐tuned neurons likely shaped by a combination of excitatory and inhibitory inputs from lower auditory centers).^87^ In the special case of bats, the IC may even read out spatial information from echolocation calls as a large proportion of neurons present delay tuning (demonstrated through playback experiments^88^, ^89^, ^90^, ^91^).

It is interesting to note that regions further from the periphery along the ascending auditory pathway seem to acquire sharper tuning curves in frequency and in sound pressure level,^92^ resulting in highly specific response functions in the IC,^93^ and also further along in the AC.^94^

Together, these findings illustrate the IC's specialization for processing various sound properties and raise the question of whether the IC could also use this information to modify and adapt properties of echolocation calls during production. Although lesioning the IC does not seem to impair the production of echolocation calls,^95^ it may play a more subtle role in controlling echolocation call parameters. When Diebold et al. transiently inactivated the IC in *E. fuscus* during free flight, bats not only performed more poorly in navigating around an obstacle but also altered several echolocation call parameters. Specifically, they showed increased call rates, longer call durations, and broader frequency bandwidths.^96^ This could either mean that bats require a functioning IC to control and adapt these aspects of echolocation calls, or that the behavioral changes reflect a compensatory mechanism to a deficit in processing spatial features of the environment. Due to its seemingly privileged position in the auditory pathway, it is likely that bats depend on sensory feedback from the IC to adjust subsequent echolocation calls.

Furthermore, the IC could play a role in social interaction and communication. When IC spike rates were recorded in *E. fuscus* during social encounters, Salles et al. found increased activity during communication calls associated with aggressive interaction, but also with calls produced during mating.^97^ That IC neurons encode spectrotemporal features of conspecifics’ communication calls had already been shown previously in *T. brasiliensis*.^98^ However, it is not clear whether IC spiking differs for self‐produced vocalizations versus calls of conspecifics.

## FOREBRAIN NETWORKS IN VOCALIZATION

### Processing of spatial and social information in the hippocampus

Beyond the midbrain, several regions in the mammalian forebrain are highly involved in spatial processing and therefore could, in the special case of bats, play a role in vocalizations—among them the hippocampus. As first described in rodents^99^ and later shown for many other mammals^100^, ^101^, ^102^ and birds,^103^ neurons in the hippocampus form place fields, becoming active whenever the animal is in a specific location within an environment.

That place cells are informed by echolocation calls was shown by Ulanovsky and Moss in *E. fuscus* when they recorded hippocampal activity while bats explored an arena. Immediately after an echolocation call, the place field of the animal's current location was clear and well localized. But as several hundreds of milliseconds passed after the call, the spatial code became less selective, resulting in larger and more diffuse place fields.^104^ The same group could also show a few years later that in periods with high call rates, hippocampal place fields are smaller and resulted in a higher resolution at close target range.^105^ A similar feature had already been described for the egocentric code in the SC.^76^ In primates and bats, hippocampal cells also code for the location to which the animal attends through visual gaze or a directional echolocation pulse.^104^, ^106^

Further experiments indicate that hippocampal oscillatory activity is modulated by echolocation calls. Theta rhythms [∼5–10 Hz], which are highly prominent in rodents during active exploration,^107^ appear in bats only intermittently in bouts,^108^ and become more frequent with rising echolocation call rates.^102^

Until now, research in the hippocampus has focused on the role of echolocation calls on spatial representations, but hippocampal activity during communication calls has not been investigated. Recent studies in *R. aegyptiacus*, however, suggest that hippocampal neurons also encode aspects of social interaction, such as the movement of a conspecific,^109^ the identity of other bats in a group setting,^110^ or even the identity of human experimenters.^111^ Relatedly, the rodent hippocampal subregion CA2 is known for its role in social interaction.^112^, ^113^ This raises the question of whether hippocampal activity is also modulated by the perception or production of social communication calls.

### Basal ganglia and dopaminergic neurons

Although the contribution of the hippocampus in vocal production is not yet resolved, studies have investigated the role of other subcortical forebrain structures; for example, the caudate nucleus (CN) in the dorsal striatum, which is part of the basal ganglia (BG), is a critical component in the motor system. When Schwartz and Smotherman investigated the expression of the immediate early gene c‐Fos (a marker for neuronal activation) in *T. brasiliensis*, they found a clear increase in expression in the CN after periods of extensive vocalization, but not when the bats had been listening only to playback calls.^114^ Additionally, oscillatory LFP activity in the CN was linked to the type of call produced in *C. perspicillata*.^115^ Specifically, beta oscillations [12–30 Hz] were predictive of the call type, exhibiting higher power before the onset of echolocation calls compared to communication calls. Simultaneously, the CN showed call‐type–dependent synchronized activity within an auditory‐selective area in the frontal cortex—the frontal auditory field (FAF, see below). This synchrony was expressed as theta coherence, a measure of phase relationship and consistency between two structures (see Glossary in Table 2), which was strongest before and during communication calls but was reduced before the onset of echolocation calls.

In a previous study, a causal role of dopaminergic neurons, which are an integral part of the BG, in vocal production had been demonstrated. After systemic injection of a dopamine‐selective neurotoxin, *T. brasiliensis* showed clear changes in their echolocation calls as amplitude, duration, and frequency bandwidth decreased. Surprisingly, similar effects were observed after the injection of a dopamine receptor agonist.^116^ This could point to a nonlinear relationship between dopamine levels and vocal production, where motor outputs are negatively affected by deviating (i.e., higher or lower) from optimum levels of dopamine activity. Alternatively, the injected neurotoxin could have temporarily increased dopamine levels before degrading dopaminergic neurons and therefore mimicked the effects of a dopamine agonist on echolocation calls. In any case, these results highlight the critical role of dopamine in vocal production and align with its well‐established function in coordinating and refining movement in other motor pathways.^117^, ^118^

### Production of emotive vocalizations via the amygdala

While the BG most likely plays a role in fine motor control of all vocalizations, communication calls in particular may feature arousing and affective elements. An area highly involved in emotion and motivation across species is the amygdala.^119^ In several bat species, it has been shown that amygdala neurons respond differentially to communication calls of conspecifics and depend on their emotional significance (*P. parnellii*;^120^ *E. fuscus* ^121^). The role of the amygdala in vocal production was probed by electrically or pharmacologically stimulating the basolateral or central nuclei in *P. parnellii*.^122^ After stimulation, bats elicited both echolocation and communication calls. Although there were no clear region‐specific boundaries regarding the call types, some topography was observed across the rostrocaudal axis, with more echolocation calls elicited after stimulation in caudal regions and more communication calls produced after rostral stimulations. The bats emitted several different types of communication calls, with the majority of evoked social vocalizations resembling calls emitted during aggressive encounters (see Figure 1B right). This is in accordance with the previously described function of the rodent amygdala in aggression and fear responses,^123^, ^124^ and highlights its complex role in various aspects of emotional behavior. Interestingly, vocalizations were emitted after a considerable latency of more than 200 ms, with longer latencies on average for echolocation calls. This points to a long signaling pathway for vocal–motor execution that could potentially run through the cortex since the amygdala shares reciprocal connections with several cortical areas (Figure 2), for example, the anterior cingulate cortex (ACC).^125^, ^126^, ^127^

### Auditory cortex involvement in sensory feedback and production

Unsurprisingly for these auditory specialists, the AC of bats is highly specialized to accommodate the demands of echolocation. In all bats that have been studied using functional neuroanatomical and electrophysiological methods, the AC is hypertrophied, exhibiting a large area dedicated to the processing of high‐frequency sounds.^128^ Similar to their subcortical counterparts, these populations are recruited during vocalizations. Across many species with different echolocation signals, AC neurons exhibit delay tuning,^129^, ^130^ which is observable in many auditory‐responsive brain regions (discussed above). In at least some species, delay‐tuned neurons appear to be spatially organized by their preferred delay, which results in a chronotopy^131^, ^132^, ^133^ that is analogous to the tonotopy—the arrangement of frequency‐tuned neurons by their preferred frequency—in primary auditory areas. This chronotopic arrangement of delay sensitive neurons is observable even in juvenile bats.^134^

The sensitivity of the AC to temporal information may enable bats to adjust vocal timing parameters. Following lesioning of the AC, Yuma myotis bats (*Myotis yumanensis*) failed to adjust their call rate when flying towards obstacles.^135^

We have discussed how neural spiking activity correlates with vocalization and the adjustment of vocal parameters. However, LFP (see Glossary in Table 2) representing a summation of synaptic activity in the AC may also provide an index of vocal‐related neuronal activity. In *C. perspicillata*, oscillations in the beta range [12–30 Hz] show higher power before the onset of a communication call compared to the pre‐vocal period of an echolocation call and can therefore be predictive of the call type that is about to be emitted.^136^ Whether this pre‐vocal beta oscillation in the AC has a specific function, such as inhibiting responses to self‐generated sounds, is still unknown.

In addition to its central role in sound processing and relevance for feedback‐dependent vocal control described above, the AC may serve to prepare the auditory network for subsequent processing of echoes through its reciprocal connections with other forebrain regions (see below).

### Neuronal activity preceding vocal onset in frontal cortical areas

In primates, the ACC has long been implicated in the voluntary control of vocalizations.^137^, ^138^ In bats, we find several indications that neuronal activity in the ACC is involved in vocal production. Electrical stimulation in the ACC of *P. parnellii* could elicit either echolocation or communication calls, depending on the area of stimulation. More anterior sites led to echolocation calls, whereas stimulation in posterior areas produced communication calls.^139^ The calls were observed with a considerable latency of approximately 100 ms after stimulation, indicative of a longer signaling pathway to the motor neurons which potentially runs through the PAG, as postulated in primates.^28^ In the ACC of *T. brasiliensis*, Schwartz and Smotherman identified increased expression of c‐Fos after extended periods of vocalization, indicative of high neuronal firing.^114^ The expression of *FOXP2*, a gene implicated in the development and learning of language, has also been demonstrated in the ACC of multiple bat species of the rhinolophid, phyllostomid, and pteropodid families.^140^, ^141^

**FIGURE 4:**
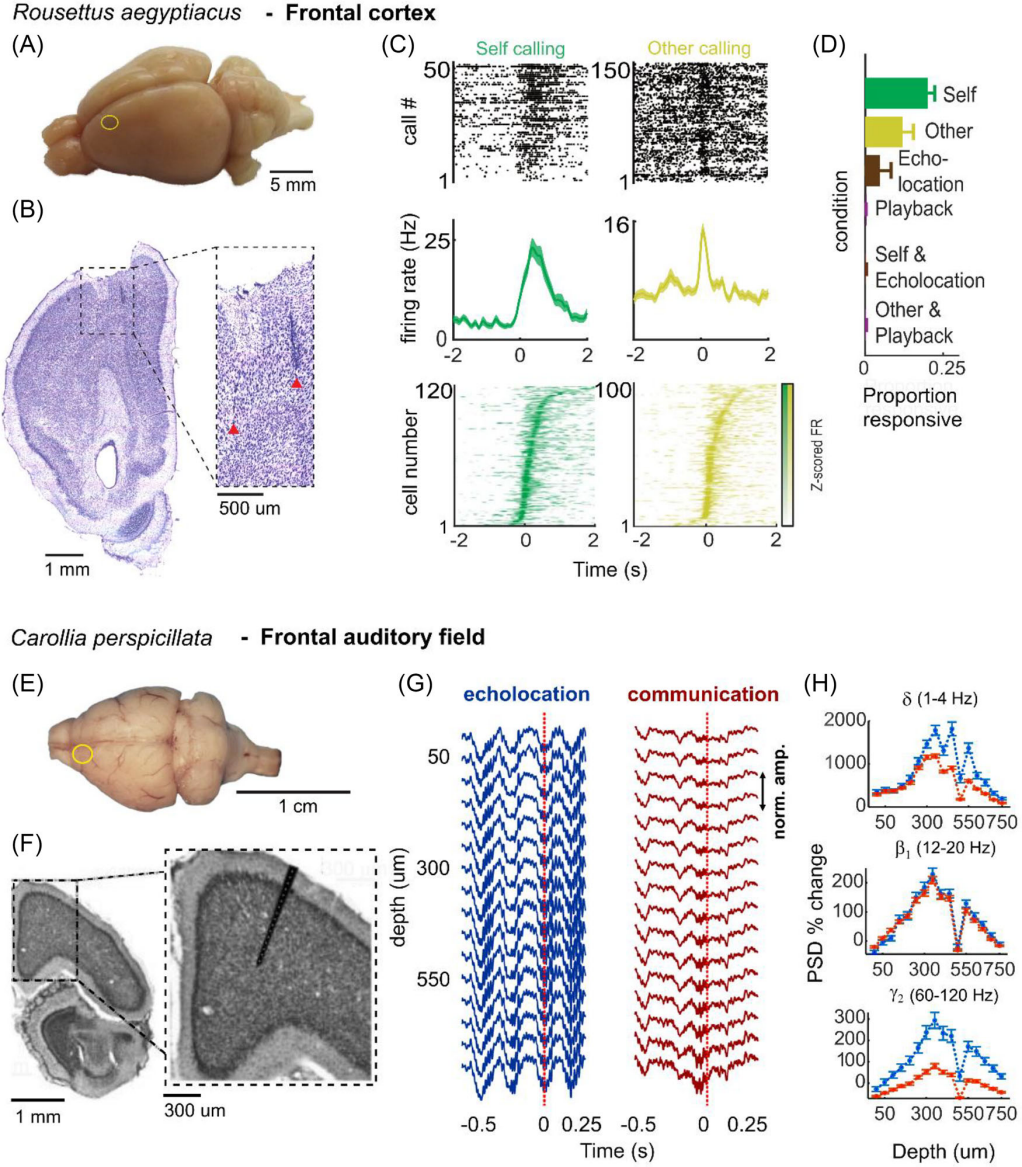
Neuronal correlates of vocalization in the frontal cortex from two different bat species. (A–D) Exemplary anatomical and electrophysiology data from *Rousettus aegyptiacus*. (A–B) Location of tetrode recording in the frontal cortex (yellow circle in (A), dashed box and inset in (B)). (C) Single unit activity during self‐produced vocalizations (green) or calls of conspecifics (yellow) during group interaction: call‐aligned raster plots (top), average firing rate (middle) of two example neurons, and *z*‐scored firing rates (bottom). (D) Average proportions of responsive neurons to different stimuli or vocal events across bats. (E–H) Exemplary anatomical and electrophysiological data from *Carollia perspicillata*. (E–F) Location of laminar probe recording in the frontal auditory field (yellow circle in (E), dashed box and inset in (F)). (G) Exemplary local field potential (LFP) traces aligned to a single echolocation call (blue) and a single social communication call (red) across cortical depth. (H) Mean change in the power spectral density in representative LFP bands during the pre‐vocal period (0.5–0 s) of an echolocation (blue) or a communication call (red) compared with baseline (steady state) activity. *Source*: (A–D) Figure reproduced from Ref. 142 with permission from AAAS; (E–H) Figure reproduced with permission from Ref. 136.

An increasing body of work has also implicated frontal cortical areas beyond the ACC in vocal production. For example, a recent study on *R. aegyptiacus* recorded neuronal firing rates in dorsal parts of the frontal cortex and identified cells that were selectively active either to self‐produced communication calls or to the vocalizations of conspecifics, with very little overlap between the populations (Figure 4).^142^ A subsequent study investigated the motor cortex of *R. aegyptiacus* ^30^ and recorded neurons specifically in an area where previously tongue, jaw, and nose movements in response to electric stimulation had been reported,^143^ and which they termed the orofacial motor cortex. In humans, damage to the orofacial motor cortex can strongly affect the ability to speak,^28^, ^68^ and in the Alston's singing mouse (*Scotinomys teguina*), a rodent from Central American rainforests that engages in elaborate territorial counter‐singing and turn‐taking behavior, inactivation of the orofacial motor cortex affects singing response to a conspecific's playback.^144^ In *R. aegyptiacus*, neurons in this region fired during or even before self‐produced spontaneous vocalizations but were less active to the auditory stimuli of other calling bats.^30^ Interestingly, the motor cortex seems to be able to bypass the vocal motor pathway through the PAG and can target the NA in the medulla directly, as demonstrated through neural tracing in this study on *R. aegyptiacus*. A similar connection has also been suggested in a recent preprint in *C. perspicillata*.^145^

Multiple studies, including from our research group, have focused their investigations on the FAF, an area in the frontal cortex located at the anterior sulcus, which responds to pure tones and complex sounds, as demonstrated in *P. parnellii*,^146^, ^147^ *C. perspicillata*,^148^, ^149^ and *T. brasiliensis*.^150^ This brain region is reciprocally connected to the AC (Figure 2)^147^, ^151^ but also receives direct input from the suprageniculate nucleus in the thalamus as part of a fast, noncanonical, and non‐lemniscal auditory pathway in which the IC and AC are bypassed.^147^, ^152^ As shown for *P. parnellii*, the FAF also sends direct projections to the deep layers of the SC and therefore targets a critical region in the vocal production network,^147^ as touched upon above.

During vocal production in *C. perspicillata*, oscillatory LFP activity in the FAF can be highly predictive of the type of vocalization the animal is about to emit. Oscillatory power in the delta range [1–4 Hz] and in the gamma range [30–120 Hz] is higher before the onset of an echolocation call than before the onset of a communication call (Figure 4).^115^, ^136^ Additionally, the FAF also synchronizes its activity with other cortical regions in a call‐type‐dependent manner. Simultaneous neuronal recordings in the FAF and AC could show that up until the onset of any vocalization, gamma and delta rhythms in the FAF are predictive of oscillations in the AC, indicative of a top‐down information flow from the FAF to AC. But at the moment an echolocation call is emitted, this directionality reverses as now delta oscillations in the AC lead those oscillations in the FAF. This reversal does not, however, take place after a communication call is produced, in which case delta waves in the FAF continue to predict AC activity.^136^ These results are highly suggestive that auditory and frontal areas operate in concert to direct vocal production and auditory feedback, particularly considering that such patterns are specific to active vocalization and do not occur during passive listening. After an echolocation call, the FAF requires input from the AC, which may transmit critical auditory information on echo delays^133^, ^153^ among other sensory cues.

Together, these studies indicate that frontal cortical areas are candidate structures for executive vocal control, as the responses of neural populations in these regions presage the structure and type of vocalizations before they are emitted. But despite the number of experiments that have been performed over recent years, a comparison across frontal cortical regions and across bat species is difficult because the terminology and the homology to brain regions in other mammalian species, and even between bat species, have not been consistently defined. The first study that described the FAF in bats also identified a direct projection coming from the mediodorsal nucleus of the thalamus.^147^ In other mammals, especially in rodents and primates, the cortical target of this thalamic projection is known as the prefrontal cortex, and this connection has often been described as a defining feature of prefrontal cortical areas, although not undisputed.^154^, ^155^, ^156^ According to this definition, the FAF could be considered part of the bat prefrontal cortex. Functionally, the prefrontal cortex is believed to select and guide adaptive behavior based on the current context,^156^, ^157^, ^158^ which in bats could extend to aspects of vocal control. But to what extent the FAF is indeed part of a potential bat prefrontal cortex and what other cortical areas can be included in this definition have not yet been explored. Relatedly, it is unclear whether previous studies investigating, nominally, the FAF and the ACC were in fact studying entirely distinct or overlapping regions.

A similar ambiguity surrounds the anatomical delimitation of the bat motor and premotor cortices. Some progress has been made in *R. aegyptiacus*, where motor functions were mapped in motor and somatosensory cortices through electrical microstimulation.^143^ However, this mapping may not easily generalize to other bat species that employ laryngeal echolocation, as *R. aegyptiacus* performs tongue‐based echolocation which does not engage the larynx.

Which features of vocalizations could be controlled or influenced by frontal cortical areas remains speculative at this point. Given the fast‐paced vocalization rates and changes that bats often employ during echolocation, it is likely that the cortex acts on longer time scales, for example, by initiating a syllable sequence or fixed action pattern. Once initiated, stereotypical sequences (e.g., the terminal buzz of *E. fuscus*) are likely executed independently of the frontal cortex. Studies in other vertebrates suggest that motor cortical areas are not strictly necessary for vocal production but play a role in timing and response latency (e.g., during vocal turn taking^144^, ^159^). Similarly, in bats, the frontal cortex could act as a modulator of vocalizations and give the impulse for voluntary communication calls. As some bat species exhibit a direct projection from the frontal cortex to the NA,^30^ motor commands could, however, in some cases be transmitted very quickly to downstream pattern generators and motor output areas.

## CONCLUDING REMARKS

### Are echolocation and communication calls controlled through the same vocal pathways?

In this review, we have aimed to bring together diverse reports illustrating how neural activity in bats gives rise to vocal production behavior (for an overview, see Figure 3 and Table S1). Two major points come into relief when considering this collection of findings as a whole. First, the general blueprint for motor preparatory and vocal production activity is generally conserved across taxa. The usual suspects have emerged as key nodes in the motor output system (premotor brainstem nuclei, the midbrain PAG, lemniscal and collicular structures, BG, and auditory and frontal cortices) both when we compare species within the bat clade and when looking beyond to other vocal species of primates (including humans) and rodents.

However, quite distinct from most other mammals, bats not only use their vocalization for social interactions but also to navigate through and perceive their environment. The presented evidence makes it clear how intimately vocal production and sensory processing are intertwined. In possibly all vertebrates that generate sound, and even in invertebrates such as crickets,^160^ neural mechanisms have evolved to monitor and inhibit responses to self‐generated sound. In the bat, this issue is elevated to a matter of central importance since echoes may return while the bat is still producing an echolocation call. Additionally, information gleaned from these echoes is needed to rapidly and dynamically adjust the following echolocation call, all on extremely short timescales.

In addition to these challenges, the brain must support both modes of vocalization, echolocation as well as communication. How this is achieved is still one of the pressing questions in this field. As echolocation is a means of navigation and communication calls are used to mediate social relationships, one possibility is that these vocal regimes engage at least partially distinct sets of neural populations. In this review, we have explored several brain regions that seem to be selective for echolocation, at least according to our current knowledge. Among them are the SC and some pontine nuclei, such as the PLA. Other areas, including the PAG, the amygdala, and the ACC, show evidence of some regional segregation for echolocation and communication circuits (for a review, see Ref. 161). But also within the same region, echolocation and communication calls may be represented and controlled in overlapping neuronal populations through distinct activity and connectivity patterns. This may make it possible to dynamically fine‐tune and adapt vocalizations according to the current context and behavioral state. One prime candidate for such a hub is the frontal cortex, which in bats has been investigated under the designations FAF and motor cortex. The dense interaction network of the frontal cortex with multiple other cortical and subcortical regions puts it in an ideal position for voluntary control of vocalization through integration of sensory feedback and modulation of motor patterns, selecting an adequate behavioral response for the current context. It is worth noting here that echolocation calls may additionally serve a social purpose and communicate personal information about the emitter,^162^ making the context and purpose of a vocalization more fluent than originally thought.

Currently, we can only hypothesize about how the system evolved to support both the production of echolocation and communication calls. Although not conclusive, most current evidence suggests that laryngeal echolocation evolved once in a species basal to all bats approximately 85–65 million years ago,^163^, ^164^, ^165^ and it is possible that this common ancestor already possessed a well‐developed vocal circuit for communication. Neural networks for the production of echolocation pulses may have evolved atop existing vocal pathways used for communication, alongside specialized brain regions tuned for the processing requirements of these calls. Alternatively, communication circuits could have evolved in parallel with echolocation, driven by the requirements of social interaction in large group settings. Comparing vocal production circuits in bats and other close mammalian relatives could give insight into this question.

### Limitations and future directions

What has become evident up until this point is that the subject that has captured the attention of most researchers in studying the bat brain is the echolocation behavior. While this is indeed the most unique feature of these flying mammals, considerably less attention has been paid to the neural basis of social communication. With the exception of a handful of studies (such as Refs. 39, 115, 122, 136), few have undertaken a systematic investigation into how vocal production circuits underlying echolocation and communication differ at the neuronal or circuit level. We acknowledge that it is challenging to measure reliable neural activity during intraspecific communication in naturalistic settings, though it has been achieved in the larger *Rousettus* bats, and even more so to compare it to such a different behavior as biosonar navigation in a compelling manner.

One limitation of electrophysiological recording techniques, the most commonly used in this field, is that the spatial extent of any given measurement is quite small, whereas complex sensory and motor functions are well‐understood to be achieved through the coordinated activity of many areas of the brain at once. We should therefore remain open to the possibility that some activity patterns observed in a particular region may not reflect the contribution of that region itself so much as a signal that is inherited from a different structure, upstream of the region under observation and hidden from the experimenter's field of view. This cautionary measure may be especially pertinent when studying multifaceted behavior such as the Doppler shift compensation or social communication.

Throughout the review, we have highlighted the many bat species in which vocalizations and associated neuronal activity patterns have been investigated. These species stem from multiple families and often differ largely in their habitat, guild, and social behavior, which is evidenced by their diversity in the design of echolocation calls adapted according to their ecological needs. In contrast to the more common approach in neuroscience that focuses on one or two species as model organisms for an entire clade, this diversity in bat research opens the door to investigations and comparisons of neuronal networks with regard to their evolution, their ecological niche, and how one affects the other. To date, only a handful of studies on vocal production are similar enough to allow for such a comparison across species. But from research in the auditory pathway, we know that neuronal organization in the brain can differ greatly depending on the complex interplay of the species’ behavioral adaptation and its position on the evolutionary tree. Examples of this, as discussed above, are the auditory fovea of CF bats and the chronotopic organization of delay‐tuned neurons in the AC.^133^ As not only echolocation but also communication calls vary greatly across species, we can expect a similar diversity in neural circuits specialized for vocal production. Identifying these differences and mapping them to a species’ adaptation and phylogenetic origin can play a crucial part in understanding the evolution of neuronal mechanisms that underlie the diverse behaviors across animals.

## AUTHOR CONTRIBUTIONS

Susanne S. Babl and Julio C. Hechavarría conceived the central focus of the review. Susanne S. Babl and Ava Kiai reviewed the literature and wrote the manuscript. Susanne S. Babl, Ava Kiai, Julio C. Hechavarría, and Francisco García‐Rosales revised and edited the manuscript.

## CONFLICT OF INTEREST STATEMENT

The authors declare no conflicts of interest.

## PEER REVIEW

The peer review history for this article is available at https://publons.com/publon/10.1111/nyas.15410.

## Supporting information



Table S1 Summary of findings from neuronal recordings, various manipulation techniques, and gene expression tracking in vocalizing bats.
