# Supplementary material for: Neuronal activity underlying vocal production in bats
Source: Ann N Y Acad Sci. 2025 Jul 21;1550(1):37–54. doi: 10.1111/nyas.15410 (PMC12412723; doi:10.1111/nyas.15410)
Supplement: Supplementary file 1 — Table S1 Summary of findings from neuronal recordings, various manipulation techniques, and gene expression tracking in vocalizing bats. [file NYAS-1550-37-s001.pdf]

Supplementary Table S1: Summary of findings from neuronal recordings, various manipulation techniques, and gene expression tracking in vocalizing bats.

| Brain region | Subordinate structure or area | Measures and/or perturbation of neural activity | Result | Family | Species | Reference |
|---|---|---|---|---|---|---|
| Cerebellum | | Electrophysiology | Call type dependent oscillations and spike patterns | Phyllostomidae | *Carollia perspicillata* | Hariharan et al., 2024 |
| | - | Electrical lesion | Increased variability in frequency (CF components) | Mormoopidae | *Pteronotus parnellii* | Horikawa & Suga, 1986 |
| Medulla | Nucleus ambiguus | Electrophysiology | Spike patterns correlate with vocal onsets/offsets and CF component frequency | Rhinolophidae | *Rhinolophus rouxii* | Rübsamen & Betz, 1986 |
| Pons | Parabrachial nucleus | none (gene expression tracking) | Increase in neuronal activity during vocalizations | Molossidae | *Tadarida brasiliensis* | Schwartz & Smotherman, 2011 |
| | | Pharmacological inhibition | (By GABA agonist) Lengthening of echolocation calls | Rhinolophidae | *Rhinolophus ferrumequinum* | Smotherman et al., 2006 |
| | | | (Via GABA agonists, glutamate antagonists) Lowered resting frequency of CF component, Doppler shift pitch over-compensation | Rhinolophidae | *Rhinolophus ferrumequinum* | Smotherman et al., 2003 |
| | | Pharmacological activation | Shorter expirations, fewer echolocation calls emitted within single expiration | Rhinolophidae | *Rhinolophus ferrumequinum* | Smotherman et al., 2006 |
| | | | (Via GABA antagonists, glutamate agonists) Increase in resting frequency of CF component, Doppler shift pitch compensation abolished | Rhinolophidae | *Rhinolophus ferrumequinum* | Smotherman et al., 2003 |
| | Paralemniscal area | Electrophysiology | Spiking activity correlated with vocal onset, duration Echo delay sensitivity | Rhinolophidae | *Rhinolophus rouxii* | Metzner, 1989, 1993 |
| | | Electrical stimulation | Echolocation calls elicited, Vocalization dependent spike patterns | Rhinolophidae | *Rhinolophus rouxii* | Schuller & Radtke-Schuller, 1990 |
| | | | Echolocation calls elicited | Phyllostomidae | *Phyllostomus discolor* | Fenzl & Schuller, 2002 |
| | | Pharmacological activation | Elicits echolocation calls | Phyllostomidae | *Phyllostomus discolor* | Fenzl & Schuller, 2002 |
| | | Pharmacological inactivation | When combined with PAG stimulation, echolocation call production inhibited | Phyllostomidae | *Phyllostomus discolor* | Fenzl & Schuller, 2002 |
| | Trigeminal nucleus | Pharmacological activation | (via GABA antagonist) Loss of control of “within-call” pitch, amplitude and temporal structure | Rhinolophidae | *Rhinolophus ferrumequinum* | Smotherman et al. 2003 |

| | | | | | | |
|---|---|---|---|---|---|---|
| Midbrain | Nucleus of the/ lateral lemniscus | Electrical stimulation | Purportedly “earliest” stations at which attenuated responses to self-generated calls are observed | Vespertilionidae | *Myotis grisescens* | Suga & Schlegel, 1972; Suga & Shimozawa, 1974 |
| | Periaqueductal gray | Gene expression tracking | Increased *C-Fos* expression after vocalization | Molossidae | *Tadarida brasiliensis* | Schwartz & Smotherman, 2011 |
| | | Electrical stimulation | Echolocation and multiple social communication-like calls elicited (lateral and ventro-lateral PAG) | Phyllostomidae | *Phyllostomus discolor* | Fenzl & Schuller, 2002 |
| | | | Echolocation calls no longer observed if PLA inhibited | Phyllostomidae | *Phyllostomus discolor* | Fenzl & Schuller, 2005 |
| | | | Species-specific echolocation calls | Phyllostomidae | *Phyllostomus discolor* | Suga et al., 1973 |
| | | | | Noctilionidae | *Noctilio leporinus* | Suga et al., 1973 |
| | | | | Mormoopidae | *Pteronotus parnellii* *Pteronotus suapurensis* | Suga et al., 1973 |
| | | | | Vespertilionidae | *Eptesicus fuscus, Myotis austroriparius, Myotis grisescens, Myotis lucifugus* | Suga et al., 1973 |
| | | | Communication calls elicited | Vespertilionidae | *Eptesicus fuscus* | Valentine et al., 2002 |
| | | Pharmacological activation | (By use of glutamate agonist) Communication calls elicited | Phyllostomidae | *Phyllostomus discolor* | Fenzl & Schuller, 2002 |
| | Inferior colliculus | Ablation | Echolocation calls unimpaired | Vespertilionidae | *Myotis yumanensis* | Suga 1969 |
| | | DREADD-based inactivation | Longer, broader, bandwidth, increased repetition rate echolocation calls, poorer flight and navigation behavior | Vespertilionidae | *Eptesicus fuscus* | Diebold et al. 2024 |
| | Superior colliculus | | Delay tuning sharpens with increased echolocation call rate<br><br>High echolocation rates accompany increased LFP gamma power | Vespertilionidae | *Eptesicus fuscus* | Kothari et al., 2018 |
| | | | Pre-vocal spike patterns correlate with target distance prior to echolocation call emission<br><br>Evidence for functional columnar organization: sensory neurons in dorsal layers, premotor neurons in ventral layers | Vespertilionidae | *Eptesicus fuscus* | Wohlgemuth, Kothari, et al., 2018, Sinha & Moss, 2007 |
| | | Electrical stimulation | Echolocation calls elicited | Rhinolophidae | *Rhinolophus rouxii* | Schuller & Radtke-Schuller, 1990 |

| | | | | | | |
|---|---|---|---|---|---|---|
| | | | | Vespertilionidae | *Eptesicus fuscus* | Valentine et al., 2002 |
| Basal ganglia | | Gene expression tracking | Increased *C-Fos* expression after vocalization | Molossidae | *Tadarida brasiliensis* | Schwartz & Smotherman, 2011 |
| | | Pharmacological manipulation | Manipulation of dopamine levels affects echo call amplitude, duration and bandwidth | Molossidae | *Tadarida brasiliensis* | Tressler et al., 2011 |
| | Caudate nucleus | Electrophysiology | Call type dependent LFP oscillations | Phyllostomidae | *Carollia perspicillata* | Weineck et al., 2020 |
| Cerebral cortex | Amygdala | Electrical stimulation | Echolocation (esp. caudal) and communication (esp. rostral) calls elicited | Mormoopidae | *Pteronotus parnellii* | Ma & Kanwal, 2014 |
| | Hippocampus | Electrophysiology | LFP theta bouts accompany increased echolocation call rates | Vespertilionidae | *Eptesicus fuscus* | Ulanovsky & Moss, 2007 |
| | | | Place field tuning informed by echolocation behavior | Vespertilionidae | *Eptesicus fuscus* | Ulanovsky & Moss, 2011, Wohlgemuth, Yu, et al., 2018 |
| | | | Spatial-view cells informed by echolocation behavior and behavioral state | Vespertilionidae | *Eptesicus fuscus* | Ulanovsky & Moss, 2011 |
| | Auditory cortex | Electrophysiology | Call type dependent LFP oscillations | Phyllostomidae | *Carollia perspicillata* | García-Rosales et al., 2022 |
| | | Electrical lesion | Dynamic adjustment of echolocation call rate impaired | Vespertilionidae | *Myotis yumanensis* | Suga, 1969 |
| | Anterior cingulate cortex | Electrical stimulation | Echolocation (esp. anterior) and communication (esp. posterior) calls elicited | Mormoopidae | *Pteronotus parnellii* | Gooler & O'Neill, 1987 |
| | | Gene expression tracking | Increased *C-Fos* expression after vocalization | Molossidae | *Tadarida brasiliensis* | Schwartz & Smotherman, 2011 |
| | Frontal cortex, frontal auditory field | Electrophysiology | Spike patterns differentiate own vs. conspecific social vocalizations | Pteropodidae | *Rousettus aegyptiacus* | M. C. Rose et al., 2021 |
| | | | Pre-vocal spiking activity | Pteropodidae | *Rousettus aegyptiacus* | Wirthlin et al., 2024 |
| | | | Call type dependent LFP oscillations | Phyllostomidae | *Carollia perspicillata* | García-Rosales et al., 2022; Weineck et al., 2020 |
